# The interplay between mitochondria and store‐operated Ca^2+^ entry: Emerging insights into cardiac diseases

**DOI:** 10.1111/jcmm.16941

**Published:** 2021-09-26

**Authors:** Jinliang Nan, Jiamin Li, Yinuo Lin, Muhammad Saif Ur Rahman, Zhengzheng Li, Lingjun Zhu

**Affiliations:** ^1^ Provincial Key Cardiovascular Research Laboratory Department of Cardiology The Second Affiliated Hospital Zhejiang University School of Medicine Zhejiang Province Hangzhou China; ^2^ Wenzhou Municipal Key Cardiovascular Research Laboratory Department of Cardiology The First Affiliated Hospital Wenzhou Medical University Zhejiang Province Wenzhou China; ^3^ Zhejiang University‐University of Edinburgh Biomedical Institute Haining Zhejiang China; ^4^ Clinical Research Center The Second Affiliated Hospital Zhejiang University School of Medicine Hangzhou China; ^5^ Department of Neurology Research Institute of Experimental Neurobiology The First Affiliated Hospital Wenzhou Medical University Zhejiang Province Wenzhou China

**Keywords:** cardiac hypertrophy, diabetic cardiomyopathy, endoplasmic reticulum, ischaemia‐reperfusion injury, mitochondria, store‐operated Ca^2+^ entry

## Abstract

Store‐operated Ca^2+^ entry (SOCE) machinery, including Orai channels, TRPCs, and STIM1, is key to cellular calcium homeostasis. The following characteristics of mitochondria are involved in the physiological and pathological regulation of cells: mitochondria mediate calcium uptake through calcium uniporters; mitochondria are regulated by mitochondrial dynamic related proteins (OPA1, MFN1/2, and DRP1) and form mitochondrial networks through continuous fission and fusion; mitochondria supply NADH to the electron transport chain through the Krebs cycle to produce ATP; under stress, mitochondria will produce excessive reactive oxygen species to regulate mitochondria‐endoplasmic reticulum interactions and the related signalling pathways. Both SOCE and mitochondria play critical roles in mediating cardiac hypertrophy, diabetic cardiomyopathy, and cardiac ischaemia‐reperfusion injury. All the mitochondrial characteristics mentioned above are determinants of SOCE activity, and vice versa. Ca^2+^ signalling dictates the reciprocal regulation between mitochondria and SOCE under the specific pathological conditions of cardiomyocytes. The coupling of mitochondria and SOCE is essential for various pathophysiological processes in the heart. Herein, we review the research focussing on the reciprocal regulation between mitochondria and SOCE and provide potential interplay patterns in cardiac diseases.

## 1 INTRODUCTION

Calcium orchestrates the Ca^2+^‐rich organelles (endoplasmic reticulum [ER] and mitochondria) and Ca^2+^‐related signalling pathways involved in physio‐ and pathological processes of eukaryotic cells.^1^ The variations in its intracellular concentration represent a major determinant that controls related signalling pathways, resulting in the modulation of cellular secretion, motility, proliferation, cell division, excitation/contraction coupling, and apoptosis.^2^, ^3^ Calcium is particularly vital for maintaining systolic and diastolic function through excitation‐contraction coupling,^4^ and cytoplasmic Ca^2+^ ([Ca^2+^]c) is important for the activity of related signal pathways and nuclear gene expression in cardiomyocytes.^5^, ^6^ Disturbances or deficits in Ca^2+^ signalling are closely related to hypertrophic cardiomyopathy, heart failure, diabetic cardiomyopathy, and cardiac ischaemia‐reperfusion injury (IRI).

Since cells use Ca^2+^ as a messenger to drive many fundamental processes, it is necessary to clarify the Ca^2+^‐entry mechanisms. Voltage‐gated Ca^2+^ channels (VGCCs) are the most studied plasma membrane (PM)‐anchored channels, and store‐operated Ca^2+^ entry (SOCE) channels on PM have received increasing attention.^7^, ^8^ Ca^2+^ flows into electrically excitable and non‐excitable cells via VGCC and SOCE, respectively. Cardiomyocytes are excitable cells, and previous studies have mainly focussed on their VGCCs. Recently, SOCE has received increased attention in cardiomyocytes; SOCE‐dependent Ca^2+^ signalling is crucial for the development of the heart and the pathological processes of cardiovascular diseases.^8^, ^9^, ^10^

L‐type VGCC, a plasma membrane‐localised voltage‐dependent calcium channel, is critical for the excitation‐contraction coupling of the heart.^11^ In cardiomyocytes, the dihydropyridine receptor (DHPR) is located near the ER‐anchored protein, sarcoplasmic reticulum ryanodine receptor 2 (RyR2).^11^ This spatial proximity allows the conversion of the depolarized plasma membrane to the release of Ca^2+^ from ER stores.^12^ During the activation of voltage‐dependent calcium entry, the periodic oscillation of Ca^2+^ concentration in the cytoplasm mediates the contraction and relaxation of cardiomyocytes. The intracellular calcium concentration is precisely regulated to avoid cellular injury. The calcium balance is maintained by Ca^2+^‐permeable channels (mainly DHPR and RyR2), calcium pumps, and Na^+^‐Ca^2+^ exchangers (regulating intracellular Ca^2+^ concentration via exchange of Ca^2+^ and Na^+^).^12^, ^13^, ^14^, ^15^ The malfunction of one machinery can be compensated for by others, helping maintain a physiological Ca^2+^ level during the activation of voltage‐dependent calcium entry. Calcium oscillations during excitatory contractile coupling of cardiomyocytes do not change the overall intracellular calcium concentration. Compared with DHPR/RyR2, SOCE mediates sustained intracellular calcium influx resulting in a moderate increase in intracellular calcium concentration.^16^ In the heart, changes in the overall calcium concentration in the cytoplasm are directly related to mitochondrial function and dynamic changes; therefore, it is necessary to clarify the interaction pattern between SOCE and mitochondria rather than DHPR/RyR2.^17^, ^18^

**FIGURE 1:**
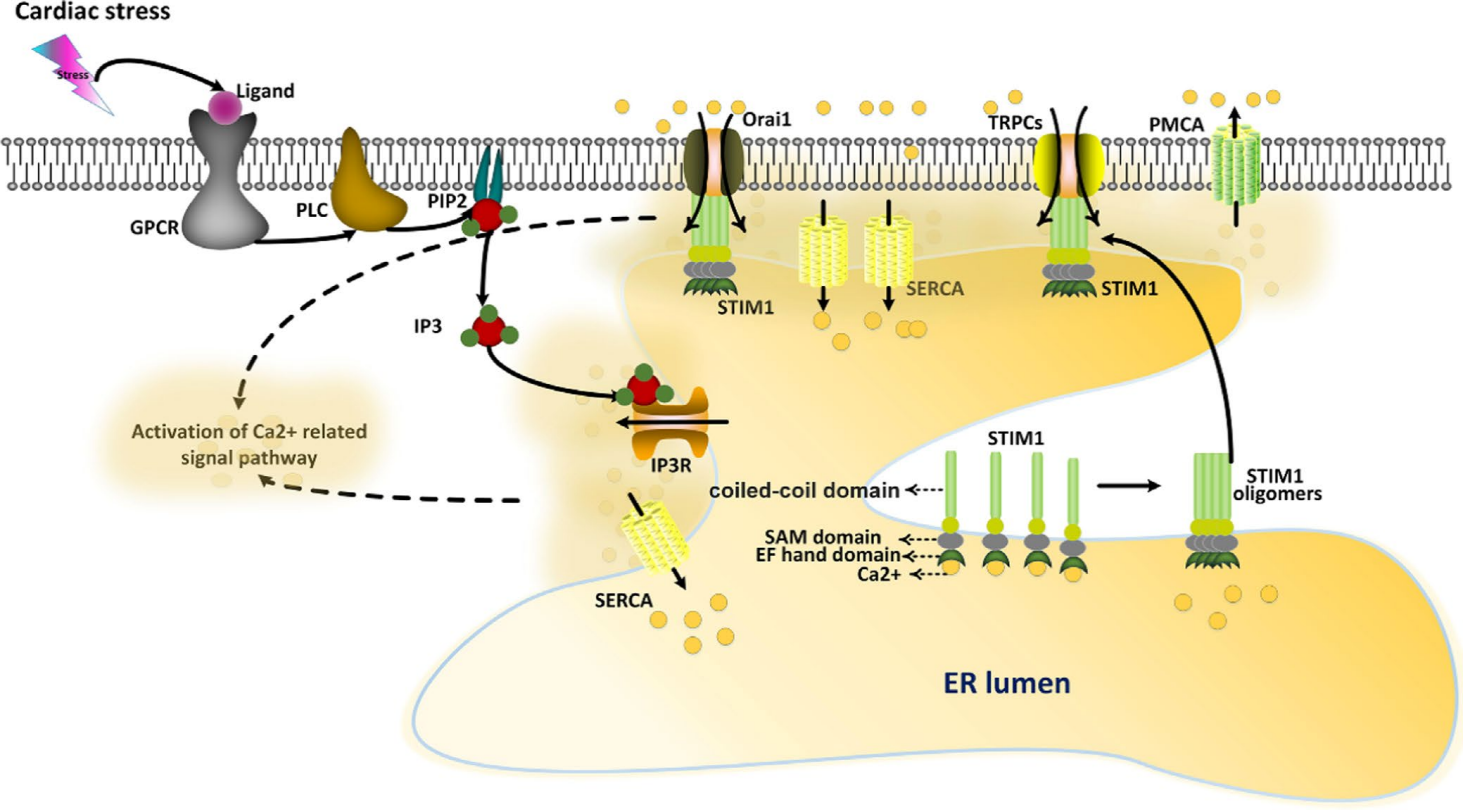
STIM1 oligomerizes to activate SOCE in the heart upon stress stimulation. Upon agonist stimulation of its receptor (GPCRs), Phospholipase C (PLC) is activated by GPCRs and subsequently promote the liberating of IP3 from PIP2. IP3 binds to its receptor (IP3R) to facilitate Ca^2+^ release from the endoplasmic reticulum ER lumen. These events lead to decreased Ca^2+^ concentration in ER and ensuing oligomer formation of STIM1; then, STIM1 oligomers bind to SOCE channels, such as Orai1, to promote Ca^2+^ influx. SERCA pumps are also concentrated around STIM1 oligomers for re‐uptaking influx Ca^2+^ through SOCE channels. Such an organization will avoid massive diffusion of Ca^2+^ ions in the cytosol, and Ca^2+^ are re‐uptaken by the pumps as soon as they reach the cytosol

Inositol 1,4,5‐triphosphate (IP3) activation is critical for ER Ca^2+^ release, causing persistent SOCE events and continuous cytoplasmic Ca^2+^ entry. IP3 is activated in the heart under pathological conditions. G protein‐coupled receptors, the plasma membrane receptors, are activated when binding to endothelin I and angiotensin II (two hormones that contribute to cardiac pathological changes), leading to phospholipase C‐dependent release of IP3 from phosphatidylinositol 4,5‐bisphosphate; then, IP3 binds to the IP3 receptor (IP3R), which is an ER Ca^2+^‐release channel, to activate it^19^, ^20^, ^21^; the interaction of IP3 and IP3R elicits Ca^2+^ depletion from the ER lumen to the cytosol^22^ (Figure 1).

In addition to their central role in metabolism, mitochondria are Ca^2+^ storage organelles; this function is enabled by the mitochondrial Ca^2+^ uniporter (MCU).^23^, ^24^, ^25^, ^26^ SOCE is related to MCU activation and calcium accumulation in the mitochondrial matrix, leading to dramatic changes in mitochondrial function such as adenosine triphosphate (ATP) production, reactive oxygen species (ROS) production, location, motility, fission, and fusion.^25^, ^27^, ^28^, ^29^, ^30^, ^31^, ^32^, ^33^ Conversely, these alterations in mitochondrial function are capable of regulating SOCE activity. The coupling of mitochondria and SOCE is essential for sustaining the constitutive activation of downstream signal pathways.^34^

Accumulating evidences supports the existence of reciprocal regulation between mitochondria and SOCE in the physiological and pathological processes of the heart.^35^, ^36^, ^37^, ^38^, ^39^ In this review, we aim to show the potential interplay patterns between SOCE and mitochondria in the heart, thus providing a theoretical basis for future studies.

## 2 MOLECULAR REGULATION OF SOCE

SOCE enables Ca^2+^ entry from the extracellular space into the cytosol.^40^, ^41^ The SOCE machinery includes three major components: stromal interaction molecule 1 (STIM1), transient receptor potential channels (TRPCs), and calcium release‐activated calcium channel protein (Orai). Based on the properties of biophysics, electrophysiology, and biochemistry, SOCE can be divided into two subgroups: Ca^2+^ release‐activated Ca^2+^ channel (CRAC) is a Ca^2+^ selective channel formed by Orai1^42^; store‐operated channels (SOC) are non‐selective cation channels formed by TRPCs.^43^ CRAC and SOC are both implicated in cardiovascular pathological processes.

Orai and TRPCs are PM‐anchored proteins; the channels formed by these two proteins induce Ca^2+^ influx from extracellular space to cytosol. Orai1, Orai2, and Orai3 are subfamily members of Orai. Orai proteins form Ca^2+^‐selective (PCa/PNa >1000) channels.^44^ Orai1 has been well studied; it forms a hexamer upon activation, but remains as a homodimer or homotetramer in its resting state.^45^, ^46^, ^47^, ^48^, ^49^ However, there is a lack of studies on SOCE regulation by Orai2 and Orai3. TRPCs form non‐selective (PCa/PNa <10) cation channels, mediating inward flows that contain cations including Ba^2+^, Na^+^, Sr^2+^, and Ca^2+^. TRPCs contain six subgroups: TRPC1, TRPC3, TRPC4, TRPC5, TRPC6, and TRPC7.^43^

SOCE events are activated by the discharge of Ca^2+^ from the ER.^50^ The key point here is how SOCE channels respond to the decreased Ca^2+^ concentration in the ER; the answer to this question was obscure until the discovery of STIM1, an endoplasmic Ca^2+^ depletion sensor protein. STIM1 oligomerization is initiated upon the release of Ca^2+^ from the ER lumen. STIM1 and STIM2 are two subfamily members of the STIM. STIM1 has been well studied and has been proven to be a Ca^2+^ sensor.^51^, ^52^ However, the physiological role of STIM2 is much less clear.^53^

STIM1 is a transmembrane protein of the ER; it consists of an intra‐ER EF‐hand domain, a transmembrane domain, a sterile α‐motif domain, an extra‐ER distal lysine‐rich cytosolic tail, and an extra‐ER coiled‐coil domain. In the resting state (~500 μM Ca^2+^ in the ER), Ca^2+^ inhibits STIM1 when binding to its EF‐hand domain.^54^, ^55^ Calcium‐binding in the EF‐hand domain can inhibit STIM1 oligomer formation, whereas the reduction of Ca^2+^ content in the ER can promote STIM1 oligomer formation and expose the activating region within the coiled‐coil domain.^56^ Oligomerized STIM1 binds to SOCE channels (Orai) at the ER‐PM junction site to elicit Ca^2+^ influx^55^ (Figure 1). In short, in response to decreased Ca^2+^ levels in the ER lumen, STIM1 oligomerizes and interacts with SOCE channels to elicit Ca^2+^ influx. Ca^2+^ entry will persist as long as Ca^2+^ levels remain low in the ER.^57^

During SOCE, calcium removed from the ER can be replenished again with SOCE‐mediated Ca^2+^ influx. SOCE increases Ca^2+^ concentration in the cytoplasm, thereby activating calcium‐dependent signalling pathways. The ER membrane‐localised sarco/endoplasmic reticulum Ca^2+^‐ATPase (SERCA) uptakes Ca^2+^ from the cytosol to the ER lumen. Following SOCE activation, SERCA pumps are aggregated around STIM1 oligomers; SERCA re‐uptake Ca^2+^ ions as soon as they cross the SOCE channels and reach the cytosol, avoiding the diffusion of Ca^2+^ ions in the cytosol and efficiently refilling the ER Ca^2+^ store (Figure 1). Some Ca^2+^ ions diffuse into the cytosol to mediate the activation of related signalling pathways.

Both the Na^+^/Ca^2+^ exchanger (NCX) and plasma membrane Ca^2+^ ATPase (PMCA) are capable of inducing Ca^2+^ extrusion from the cytosol.^58^ NCX has low Ca^2+^ affinity but a high capacity for Ca^2+^ transport, whereas PMCA exhibits the opposite properties.^59^ Large cytosolic Ca^2+^ variations (both amplitude and content changes) have been observed when cardiomyocytes undergo excitation‐contraction coupling, mainly achieved by dynamic regulation of NCX, but not by PMCA.^60^ By comparison, PMCA counteracts the mild and persistent Ca^2+^ elevation elicited by SOCE in the cytosol. Therefore, PMCA activation is the main factor that mediates Ca^2+^ extrusion during SOCE.^61^

## 3 RECIPROCAL REGULATIONS BETWEEN MITOCHONDRIA AND SOCE

SOCE induces Ca^2+^ influx from regions adjacent to Ca^2+^ entry sites with high Ca^2+^ concentrations, referred to as calcium concentration microdomains (CCMs).^62^ When the Ca^2+^ level in CCMs is high, it inhibits the activity of SOCE channels via a process called Ca^2+^‐dependent SOCE inactivation. This property of the SOCE channel is crucial for the interplay between SOCE and mitochondria.

Mitochondrial function is closely related to the activity of SOCE.^63^, ^64^ Mitochondrial depolarization by FCCP mediates STIM1 clustering, resulting in enhanced store‐operated channels activity.^65^ Mitochondria support most of the physiological actions of cells by producing ATP.^66^ ATP can buffer Ca^2+^, relieving the inhibitory effect of incoming Ca^2+^ on SOCE activity.^67^ We can thus infer that the metabolic status of mitochondria fine‐tunes intracellular ATP content, which is in turn key to determining SOCE activity. Various diseases, including cardiovascular, neurological, and immune diseases, exhibit changes in both Ca^2+^ signalling and ROS production in cells.^68^, ^69^, ^70^, ^71^, ^72^ Both Ca^2+^ signals and ROS are mainly handled by the mitochondria and are linked to SOCE activity.^73^, ^74^, ^75^, ^76^ Thus, mitochondria regulating SOCE activity may also be dependent on mitochondrial Ca^2+^ ([Ca^2+^]m) handling and ROS generation. Moreover, location, motility, and dynamics of mitochondria are also determinants of SOCE activity.^30^, ^31^, ^33^

Conversely, SOCE plays a vital role in regulating mitochondria.^27^, ^28^ SOCE‐induced Ca^2+^ influx can affect the concentration of [Ca^2+^]m and subsequently modulate mitochondrial energy metabolism (ATP production) and mitochondrial stress response (ROS generation). MCU responds to increased SOCE activity and initiates [Ca^2+^]m uptake to accumulate Ca^2+^ within the mitochondrial matrix^77^, ^78^; thereafter, increased [Ca^2+^]m concentration stimulates oxygen consumption to accelerate ATP production and ROS generation.^79^ SOCE can affect mitochondrial production of ATP and ROS, and vice versa. Under certain conditions, feedback loops can be formed between them to mediate cytopathic processes.

### 3.1 Spatial distributions of CCMs and mitochondria in cardiomyocytes determine the activity of SOCE

**FIGURE 2:**
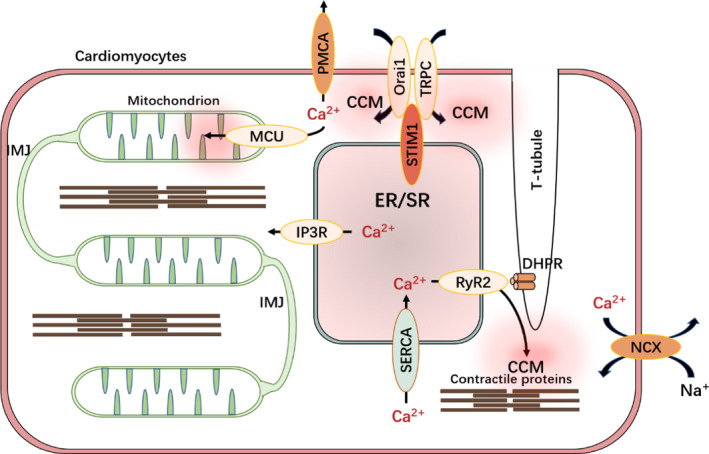
The spatial distribution of the mitochondrial network and CCM in cardiomyocytes. In cardiomyocytes, mitochondria are separated by muscle filaments and are mainly divided into sub‐PM mitochondria and interfibrillar mitochondria. But mitochondria can still be connected through inter‐mitochondrial junction (IMJ) to form a mitochondrial network. IMJ promotes the exchange of metabolites and mitochondrial DNA among independent mitochondria and inhibits the transmission of stress in the mitochondrial network. Sub‐PM mitochondria are close to the SOCE channels and mediate Ca^2+^ uptake through the activation of MCU. There are two different types of CCM in cardiomyocytes: The first CCM is mediated by DHPR/RyR2 and is located near the muscle filament deep in the cytoplasm, promoting the contraction of cardiomyocytes. DHPR/ RyR2‐mediated CCM can be rapidly dissipated through SERCA and NCX activation, thus allowing cardiomyocytes to enter the diastolic phase. DHPR/RyR2‐mediated CCM is consistent with excitatory contraction coupling of cardiomyocytes and occurs periodically. Abbreviations: CCM, calcium concentration microdomains; DHPR, dihydropyridine receptor; IMJ, inter‐mitochondrial junction; MCU, mitochondrial Ca2+ uniporter; NCX, Na+/Ca2+ exchanger; PMCA, plasma membrane Ca2+ ATPase; RyR2, reticulum ryanodine receptor 2; SERCA, sarco/endoplasmic reticulum Ca2+‐ATPase

The Ca^2+^ channels (DHPR) in the T‐tubule membrane come very close to SR localised Ca^2+^ release channels (RyR2) to form a calcium release unit (CRU) (Figure 2) that induces the formation of CCMs to activate myofilaments.^80^ Both CRU and SOCE channels can induce the formation of CCMs in cardiomyocytes.^81^ CRU‐elicited CCMs are located in the deep cytosol, far from the PM‐localised SOCE channels (Figure 2). Therefore, sub‐PM‐localised CCMs (induced by SOCE activation) contribute to SOCE inhibition. CCMs mediated by CRU can dissipate rapidly in two ways: NCX‐mediated Ca^2+^ transport from the cytoplasm to the extracellular matrix, or SERCA‐mediated Ca^2+^ transport from the cytoplasm to the ER lumen.^80^ The rapid dissipation of CCMs mediated by CRUs is necessary to convert cardiomyocytes from systole to diastole. SOCE‐mediated Ca^2+^ influx is relatively more persistent, allowing SOCE‐related CCM to inhibit SOCE channels.

Conventionally, it was believed that mitochondria, localised in the vicinity of CCMs, directly regulated SOCE activity. Following the SOCE event, mitochondria were thought to take up Ca^2+^ ions near CCMs to buffer them and prevent Ca^2+^‐dependent inactivation of SOCE. However, this proposed mechanism was challenged after the discovery of the STIM proteins. Activation of SOCE channels is initiated and maintained by protein‐protein interactions between ER membrane‐anchored proteins (STIM) and PM‐anchored proteins (Orai1), together with the ER and PM.^82^, ^83^ The evidence for direct interaction between STIM1 and TRPCs is insufficient, but studies have shown that STIM1 mediates TRPCs‐dependent activation of SOCE.^42^, ^83^ The distance between ER and PM in this condition has been found to range between 8 and 14 nm^84^, ^85^; therefore, the space between the ER and PM upon SOCE activation is not able to accommodate a mitochondrion. However, evidence still exists that the SOCE event promotes [Ca^2+^]m uptake and, in turn, sub‐PM mitochondria are capable of modulating SOCE activity.^86^, ^87^, ^88^ Therefore, the specific mechanism of the interplay between sub‐PM mitochondria and SOCE should be further explored and redefined.

Focussed ion beam scanning electron microscopy has helped to achieve 3D rendering of cardiac mitochondria. Using this approach, the inter‐mitochondrial junction has been found to exist between two adjacent mitochondria, enabling rapid communication and distribution of potential energy through the cell^89^ (Figure 2). A proactive mechanism involving the separation of dysfunctional mitochondria from the mitochondrial network allows mitochondrial damage to be confined.^90^ Due to the paucity of research regarding Ca^2+^ diffusion between individual mitochondria via inter‐mitochondrial junctions, it is still not clear whether the whole mitochondrial network can freely share the Ca^2+^ ion. Presumably, as with the segregation of mitochondrial damage, SOCE‐related mitochondrial Ca^2+^ uptake may also be limited without spreading throughout the mitochondrial network (Figure 2).

Based on the distance between mitochondria and the PM, mitochondria are divided into two subgroups: sub‐PM mitochondria (close to SOCE channels) and interfibrillar mitochondria (far from SOCE channels) in cardiomyocytes.^91^ Sub‐PM mitochondria are more likely to interact with SOCE but not with interfibrillar mitochondria. Nevertheless, there is evidence of regulation between the interfibrillar mitochondria and SOCE. The proposed mechanism of interplay between SOCE and interfibrillar mitochondria in cardiomyocytes is as follows: the ER uptakes the incoming Ca^2+^ upon SOCE activation and transports those Ca^2+^ ions to the interfibrillar mitochondria by directly interacting with interfibrillar mitochondria; these in turn can continuously uptake Ca^2+^ through opened SOCE channels and export them through IP3Rs, thus affecting Ca^2+^ uptake of mitochondria in deep cytosol.^92^ In contrast, interfibrillar mitochondria can modulate SOCE activity. Research has confirmed that the disruption of mitochondrial potential with antimycin A/oligomycin, rotenone/oligomycin, and uncouplers reduces the amplitude of SOCE.^93^, ^94^, ^95^ The mitochondrial potential is correlated with increased SOCE activity, suggesting that fully functional mitochondria are essential for SOCE activity regardless of mitochondrial localization.^96^ However, the specific mechanism underlying these observations remains unclear. It is tempting to speculate that mitochondria‐released metabolites and byproducts of cellular respiration may disperse to the vicinity of SOCE channels and subsequently influence their activity.

### 3.2 Crosstalk between mitochondria and SOCE

#### 3.2.1 Mitochondrial Ca^2+^ transporters and SOCE activity

**FIGURE 3:**
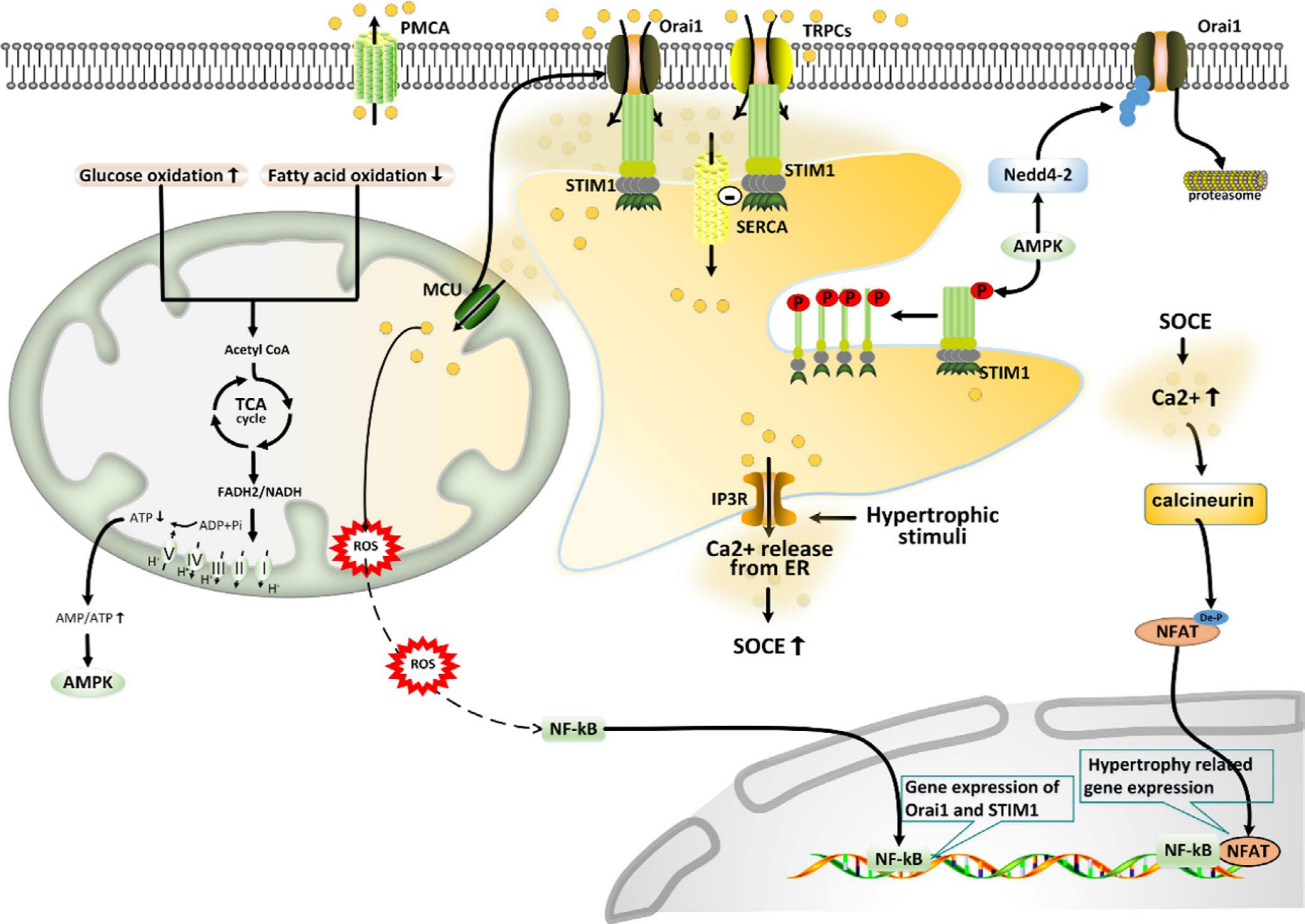
Mitochondria are closely related to SOCE during the development of cardiac hypertrophy. Hypertrophy related cardiac stress promotes the opening of IP3R, then releases Ca^2+^ from ER to initiate SOCE. SOCE‐induced Ca^2+^ influx promotes calcineurin‐dependent de‐phosphorylation (De‐p) of NFAT; this calcineurin‐NFAT signalling upregulates the expression of hypertrophy related genes. Positive feedback between mitochondria and SOCE may exist due to NF‐kB activation: mtROS activate NF‐kB to increase the transcriptional activity of Orai1 and STIM1, in turn, improved SOCE machinery further elevate mitochondrial Ca^2+^ uptake to generate mtROS. Diminished FAO and enhanced glucose oxidation cause inefficiency of the TCA cycle, leading to a decrease in ATP production and the ensuing increase in AMPK activation. AMPK negatively regulated SOCE activation through STIM1 phosphorylation and Nedd4‐2 dependent Orai1 degradation. NF‐kB facilitates whilst AMPK attenuates SOCE activity, suggesting that the regulatory pattern of mitochondria on SOCE activity is biphasic in hypertrophic heart. De‐P denotes de‐phosphorylation; P denotes phosphorylation

Since the mitochondrial inner membrane is impermeable to Ca^2+^, calcium depends on transporters to accumulate in the mitochondria. MCU, a Ca^2+^ transporter, can be activated by increased [Ca^2+^]c (cytosolic Ca^2+^) and subsequently uptake Ca^2+^ from the cytoplasm to the mitochondrial matrix; uncoupling protein 2 (UCP2) is also a Ca^2+^ transporter on the mitochondrial inner membrane. Their specific roles in Ca^2+^ handling differ. MCUs can respond well to increased Ca^2+^ levels regardless of the source of Ca^2+^, whereas UCP2 uptake from the intracellular released Ca^2+^ but not that of incoming through SOCE channels.^97^, ^98^, ^99^, ^100^ However, both are essential for mediating SOCE activity^25^, ^92^, ^101^, ^102^ (Figure 3). Ruthenium red treatment and MCU knockdown can effectively reduce serum‐ or thapsigargin‐induced SOCE.^102^ MCU‐ or UCP2‐knockdown hampers STIM1 oligomerization and subsequently inhibits SOCE upon IP3‐generating agonist treatment.^25^ In short, mitochondrial Ca^2+^ transporters (MCU and UCP2) are essential for maintaining SOCE activity.

#### 3.2.2 SOCE promotes MCU‐dependent mitochondrial calcium uptake and thus regulates mitochondrial function

It is known that Ca^2+^ uptake transporters (MCU and UPC2), Ca^2+^ extrude transporters, and Ca^2+^‐H^+^‐ exchanger (Letm1) are essential for maintaining [Ca^2+^]m homeostasis. SOCE can mediate the upregulation of MCU, thereby controlling mitochondrial Ca^2+^ uptake and regulating mitochondrial metabolism.^90^, ^103^, ^104^ There is no evidence showing that other transporters such as UCP2, Na^+^‐Ca^2+^ exchanger, and Ca^2+^‐H^+^ exchanger respond to the SOCE event.

SOCE promotes the activation of MCU that facilitates [Ca^2+^]m uptake.^97^, ^98^ MCU‐dependent [Ca^2+^]m uptake is essential for mitochondrial ROS (mtROS) generation. A study has shown that MCU is associated with mtROS production, thereby promoting cellular skeletal response during wound healing in Caenorhabditis Elegans.^105^

MCU‐dependent [Ca^2+^]m uptake regulates ATP production via oxidative phosphorylation. In mitochondria, calcium ions activate three key enzymes related to the TCA cycle: pyruvate dehydrogenase, ketoglutarate dehydrogenases, and isocitrate dehydrogenases.^106^, ^107^, ^108^ These three dehydrogenases respond to increased Ca^2+^ concentration in the mitochondrial matrix to increase NADH availability, drive electrons to flow through the respiratory chain, and ultimately enhance ATP synthesis. In addition, the SOCE‐mediated increase in cytosolic Ca^2+^ ([Ca^2+^]c) enhances the activity of aralar1 and citrin (two inner mitochondrial membrane‐anchored aspartate/glutamate exchangers) to accelerate the supply of α‐ketoglutaric acid (α‐KG) to the tricarboxylic acid cycle (TCA cycle), and ultimately promote ATP synthesis.^109^, ^110^, ^111^

### 3.3 Mitochondrial ATP, ROS, and metabolic status influence SOCE activity

ATP is a potent Ca^2+^ buffer besides mitochondria^67^; aggregation of ATP at the regions adjacent to Ca^2+^ entry sites attenuates Ca^2+^‐dependent inactivation of SOCE. Energetic mitochondria are necessary to produce sufficient ATP to buffer Ca^2+^ in CCMs. ATP can diffuse in the space surrounding the ER‐PM junction to buffer the incoming Ca^2+^ through SOCE channels; since mitochondria are not able to reach the site of the ER‐PM junction, mitochondria exert their buffering action in an ATP‐dependent manner. Alternatively, ATP may exert its effects on SOCE activity by targeting Ca^2+^ ion pumps on either the PM or ER membrane. PMCA is a transport protein on PM and functions to remove Ca^2+^ from cells. SERCA uptakes Ca^2+^ from the cytosol, leading to Ca^2+^ accumulation in the ER lumen. SERCA and PMCA are powered by ATP that results in a low Ca^2+^ concentration surrounding the SOCE channels to maintain SOCE activity.^92^ These findings open a new perspective regarding the role of mitochondrial metabolism in SOCE activity. They may help explain how mitochondrial inhibitors of the electron transport chain (ETC), antimycin A/oligomycin, rotenone/oligomycin, and uncouplers reduce the amplitude of SOCE.^93^, ^94^, ^95^

**FIGURE 4:**
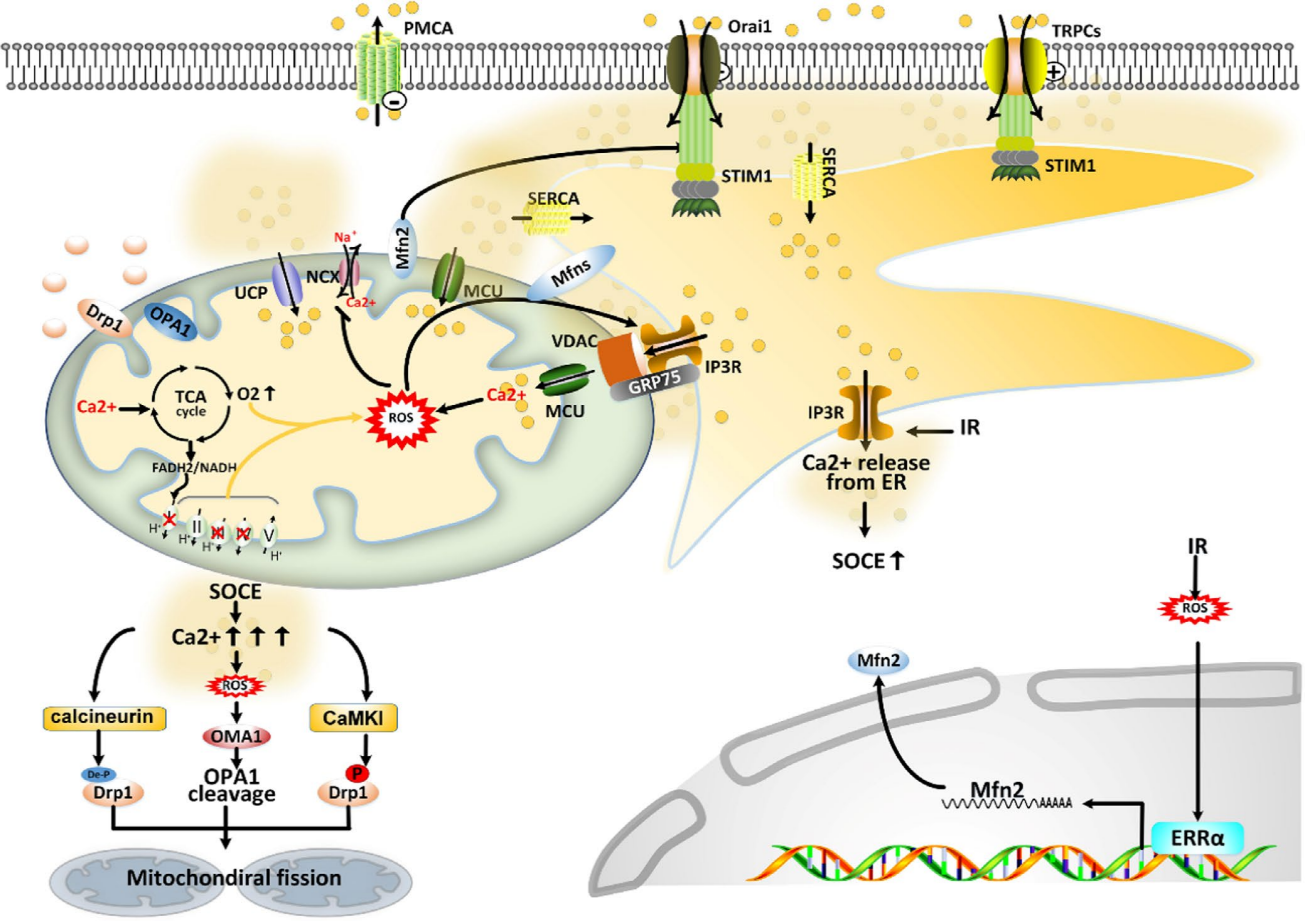
Mitochondria and SOCE are reciprocally regulated following I/R. I/R injury of the heart is characterised by Ca^2+^ overload and a burst of mtROS generation at the onset of reperfusion. For Ca^2+^ overload, SOCE is a major contributor which may result from the enhanced capability of mitochondrial Ca^2+^ uptake by MCU: mitochondrial Ca^2+^ uptake reduces the size of CCMs in the proximity of Ca^2+^ entry sites and protects SOCE channels from Ca^2+^‐dependent inactivation; mitochondria take up Ca^2+^ through IP3R to maintain a low concentration of Ca^2+^ in ER to activate SOCE. Besides, upregulated Mfn2 tether mitochondria and ER together, making IP3R and MCU at a close distance, resulting in a persistent Ca^2+^ transport from ER to mitochondria and sustained activation of SOCE. Mfn2 is also important for translocation of STIM1 to ER‐PM junction sites where STIM1 interact with and activate SOCE channels. For mtROS generation, SOCE‐induced [Ca^2+^]m accumulation (in MCU‐dependent manner) is a major contributor, which is also referred to as SOCE‐[Ca^2+^]m‐mtROS pathway. SOCE‐[Ca^2+^]m‐mtROS pathway sensitises ER to release Ca^2+^ to stabilise the opening of SOCE channels during the I/R process. Mitochondrial dynamic‐related proteins, including Drp1 and OPA1, are potential targets of downstream pathways of SOCE, leading to mitochondrial fission at the onset of reperfusion. De‐P denotes de‐phosphorylation; P denotes phosphorylation

ROS‐induced modification of STIM1 and Orai1 can affect the SOCE amplitude. Direct targets of ROS on SOCE machinery are either located in the ER matrix (cysteines C56 and C49 in STIM1) or in the extracellular space (cysteine C195 in Orai).^112^, ^113^ ROS generated by mitochondria under stress conditions are unlikely to diffuse to the regions where these modification sites are located, and direct regulation of mtROS on SOCE components, including STIM1 and Orai, seems difficult to implement.^114^, ^115^, ^116^ However, mtROS can regulate SOCE events and activity at the transcriptional level. Nuclear factor kappa B (NF‐κB) can be activated by mtROS under stress conditions; it is a transcription factor that upregulates the protein levels of SOCE components (Orai1 and STIM1).^114^, ^116^, ^117^ Moreover, mtROS also regulate SOCE activity by modulating the Ca^2+^ concentration in the ER. MtROS increase the sensitivity of IP3R to release Ca^2+^ from the ER that then keeps the ER‐Ca^2+^ level low enough to maintain SOCE activity in a STIM1‐dependent manner^118^, ^119^ (Figure 4).

Similar to ATP, pyruvic acid can also buffer Ca^2+^ in CCMs.^120^ Pyruvic acid is produced from glucose via glycolysis. Aerobic respiration (by cells with sufficient oxygen) allows pyruvic acid to take part in the TCA cycle; fermentation (by cells with insufficient oxygen) transforms pyruvic acid into lactate. Therefore, metabolic status affects the level of intracellular pyruvic acid and subsequently modulates SOCE activity.

### 3.4 Mitochondrial dynamics, mitochondrial motility, and SOCE

SOCE‐dependent regulation of mitochondrial motility in immune cells has been previously discussed.^92^ Mitochondria move to the CCMs at the Ca^2+^ entry sites during SOCE activation; then, mitochondria take up Ca^2+^ to reduce the size of CCMs and protect SOCE channels from Ca^2+^‐dependent inactivation.^30^, ^31^, ^121^ In brief, mitochondria can be immobilised at Ca^2+^ entry sites and buffer Ca^2+^ in CCMs, resulting in sustained SOCE activity. Cardiac mitochondria are tightly packed between myofibrils, thus hampering mitochondrial motility. However, mitochondrial transport does exist in cardiomyocytes, and the high concentration of intracellular Ca^2+^ regulates Miro 1/2 proteins to hamper the motility of mitochondria, implying that mitochondrial motility plays a role in SOCE activity in the heart.^122^

Mitochondrial morphology is determined by their dynamic events (fusion and fission); these events are also called mitochondrial dynamics. Several proteins were determined to be pro‐fusion proteins that induce mitochondrial fusion, such as Mfn1/2 and OPA1, that are tightly regulated by pro‐fission proteins such as Drp1 and hFis1.^123^ Mitochondrial dynamics are correlated with SOCE amplitude. Overexpression of hFis1 induces mitochondrial fission that results in decreased accumulation of sub‐PM mitochondria and subsequently attenuated SOCE activity.^86^, ^93^, ^124^ Mfn2, a pro‐fusion protein, is necessary for the trafficking of STIM1 to the ER‐PM junction to open the SOCE channels. Knockdown of Mfn2 can decrease SOCE activity, thereby reducing cellular Ca^2+^ overload.^33^

Conversely, SOCE activity has a profound impact on mitochondrial dynamics. STIM1 knockout promotes mitochondrial fusion and increases metabolic activity in mouse embryonic fibroblasts,^28^ demonstrating that SOCE is implicated in the modulation of mitochondrial dynamics and metabolism. Research data support that SOCE‐induced Ca^2+^ influx modulates mitochondrial dynamics by activating pathways that are downstream of [Ca^2+^] c. It is well established that increased [Ca^2+^]c levels activate calcineurin/Drp1 and CaMKIα/Drp1 pathways to promote mitochondrial fission.^18^, ^125^

## 4 SOCE MACHINERY IN HEART

### 4.1 SOCE is involved in cardiac development

SOCE is essential for both physiological and pathological heart regulation. The protein expression of the STIM, Orai, and TRPC subfamilies is influenced by the corresponding cell types. Determining the characteristics of the expression of these proteins in cardiac myocytes is necessary to understand the specific mechanism of myocardial SOCE. STIM1 and STIM1L (a splice variant of STIM1) transcripts are both present in neonatal cardiomyocytes but diminish with maturation. STIM1 and STIM1L have been found to be upregulated in myocardial cells under stress (e.g. phenylephrine treatment),^8^ whereas STIM2 has not been studied in the heart. The Orai protein family comprises three members (Orai1 ‐ Orai3); the most studied subfamily is Orai1 in the heart. TRPC1, TRPC3, TRPC4, and TRPC6 channels contribute to cardiac hypertrophy.^126^, ^127^, ^128^, ^129^ SOCE events are highly selective for Ca^2+^ in neonatal cardiomyocytes. We infer that Orai1 but not TRPCs mediated SOCE events that contributed to the postnatal development of cardiomyocytes.^10^ In the adult heart, STIM1‐Orai1 is implicated in SOCE activation, inducing pathologic changes in the heart.^130^

### 4.2 SOCE is involved in cardiac excitation and conduction

The regulation of Ca^2+^ is essential for the excitation and conduction of the heart. It was confirmed that STIM1 was expressed in coronary sinus cardiomyocytes (CSCs) in a tract from the sinoatrial node to the coronary sinus.^131^ The deletion of STIM1 from CSCs slowed interatrial conduction and increased susceptibility to atrial fibrillation. These data support the physiological role of the STIM1 pathway in CSCs that contributes to interatrial conduction to avoid atrial arrhythmias.^131^ SOCE may also be involved in arrhythmogenesis in mouse ventricular myocytes. Caffeine, thapsigargin, and cyclopiazonic acid treatment induce the depletion of sarcoplasmic reticulum (SR) Ca^2+^ that effectively elicits the SOCE event in mouse ventricular myocytes.^132^ When cardiomyocytes were pretreated with anti‐TRPC 1, 3, and 6 antibodies, the SOCE events and cardiac arrhythmias were significantly reduced.^132^ These observations suggest that SOCE events in mouse ventricular myocytes are mediated by TRPC channels that may be involved in cardiac arrhythmogenesis by enhancing spontaneous Ca^2+^ waves. The arrhythmogenic role of SOCE was verified by another research group using a genetic murine model of arrhythmic disease (catecholaminergic ventricular tachycardia; CPVT). They demonstrated that transient Ca^2+^ entry events comprise cardiac SOCE and can be effectively prevented by SOCE inhibition.^133^ The specific locations of SOCE events were also confirmed to be intercalated disc compartments.^133^

## 5 MITOCHONDRIA AND SOCE IN CARDIAC HYPERTROPHY

TRPCs and Orai1 are upregulated, and STIM1 is activated, during the progression of cardiac hypertrophy.^22^, ^127^, ^130^, ^134^, ^135^, ^136^, ^137^, ^138^ Since STIM1, Orai1, and TRPCs are central mediators of SOCE, directly interfering with their expressions through the corresponding knockdowns and knockouts can help to elucidate the specific role of SOCE during the progression of cardiac hypertrophy. Using these approaches, studies have confirmed that angiotensin II and endothelin‐1 enhance the SOCE event that then activates the calcineurin/NFAT signalling pathway to induce cardiac hypertrophy.^22^, ^127^, ^134^, ^135^, ^136^, ^137^, ^138^ Phenylephrine‐induced cardiomyocyte hypertrophy is mediated by SOCE‐dependent CaMKIIδ activation.^139^ Using STIM1 transgenic mice, SOCE was shown to be the major contributor to the cardiac hypertrophic response. These mice developed heart failure with hypertrophy and exhibited increased expression of hypertrophic genes.^37^ Moreover, STIM1 oligomerization promotes the formation of CCMs that are essential for remodelling cytoskeletal myofilaments during the hypertrophic progression of the heart. STIM1 knockout alleviates cardiac hypertrophic response upon pressure overload.^140^ SOCE activation is the primary cause of cardiac hypertrophy.

Hypertrophic transformation of the heart can be divided into three stages: developing hypertrophy (stage 1), compensatory hypertrophy (stage 2), and overt heart failure (stage 3).^141^ In stages 2 and 3, the reversion of metabolic preference has been observed (from fatty acid oxidation to glucose), leading to enhanced activity of AMP‐kinase (AMPK).^141^ Studies have confirmed that AMPK is associated with SOCE activity. AMPK induces the phosphorylation of STIM1 to inhibit its oligomerization and activation and subsequently attenuates SOCE activity.^142^ On the other hand, AMPK can activate Nedd4‐2 (a ubiquitin ligase) that then targets Orai1 for its degradation.^143^ Taken together, we can infer that the metabolic status in cardiac hypertrophy (stages 2 and 3) activates AMPK and subsequently negatively regulates SOCE activity (Figure 3). This may be the mechanism by which AMPK activation reduces cardiac hypertrophy.^144^

During the progression of cardiac hypertrophy, mtROS generation is moderately increased that subsequently activates its downstream signalling pathway, NF‐kB.^145^, ^146^, ^147^, ^148^, ^149^ NF‐κB is a transcription factor that enhances the expression of STIM1 and Orai1, thereby promoting SOCE activity.^114^, ^116^ Taken together, we hypothesise that mtROS‐NF‐kB promotes calcineurin/NFAT signalling pathway‐dependent cardiac hypertrophy by mediating the increase in SOCE. It should be mentioned that SOCE increases [Ca^2^ ^+^]m levels to induce mtROS‐NF‐kB signalling and, in turn, mtROS‐NF‐kB signal enhances SOCE activity to form a positive feedback loop^105^, ^116^ (Figure 3).

In hypertrophic cardiomyocytes, mitochondrial metabolic status (which activates AMPK) and mitochondrial ROS generation (which activates NF‐κB) oppositely modulate SOCE activity. AMPK attenuates and NF‐κB facilitates SOCE activity. In short, mitochondria can either promote or inhibit SOCE activity in the hypertrophic heart.

## 6 MITOCHONDRIA AND SOCE IN HEART FAILURE

### 6.1 Mitochondria and SOCE in heart failure with reduced ejection fraction (HFrEF)

Changes in the components of the SOCE machinery were observed in patients who were diagnosed with heart failure in the NYHA III‐IV class. Orai1 was decreased by 30% in failing myocardium, whilst Orai2 and Orai3 expression remained normal. The STIM2.1 levels are significantly reduced to enhance SOCE activity in end‐stage heart failure.^150^ SOCE alterations have been shown to contribute to the progression of dilated heart failure in animal models. Cardiac‐specific deletion of STIM1 in mice promotes left ventricular dilatation and decreased contractility.^140^ Cardiac STIM1 knockout and mutant STIM1 (R429C) expression exhibited mitochondrial abnormalities (>12 weeks of age) and contractile dysfunction (between 20 and 36 weeks of age).^36^, ^151^ The R140W mutation of muscle‐related coiled‐coil protein (MURC) disrupts SOCE activation and contributes to human dilated cardiomyopathy.^152^ Cardiac Orai1^+/−^ mice develop dilated cardiomyopathy that cannot compensate for the overload and results in an earlier death.^153^ Orai1^R93W^ knock‐in mice that express a non‐functional Orai1 channel protein exhibited markedly swollen mitochondria with an abnormal cristae structure in the heart.^154^ In a zebrafish model, inactivation of Orai1 resulted in the development of dilated heart failure and progressive loss of myofiber integrity.^155^ Overall, these human and animal studies strongly support the crucial role of SOCE inactivation and mitochondrial dysfunction in regulating dilated heart failure. However, further investigations are necessary to explore the specific mechanism of reciprocal regulation between SOCE and mitochondria.

In the dilated heart, the metabolic substrate preference of mitochondria converts fatty acids to glucose. Interestingly, SOCE events dramatically decrease during the transition of the heart from compensatory hypertrophy to dilated heart failure. Thorough investigations of the relationship between SOCE and mitochondrial metabolism have been conducted to elucidate their reciprocal regulation in heart failure. In the failing heart, compromised SOCE activity reduces lipid metabolism in a mitochondria‐dependent manner that is crucial for the mobilisation of fatty acids from lipid droplets and mitochondrial fatty acid oxidation; SOCE controls cAMP‐dependent induction of PGC‐1α/PPARα expression and lipolysis.^154^ Additionally, SOCE regulates mitochondrial metabolism by stimulating mitochondrial Ca^2+^ uptake, and SOCE‐dependent activation of Ca^2+^‐regulated transcription factor promotes MCU expression, in turn controlling the Ca^2+^ uptake capability of mitochondria and hence regulating mitochondrial metabolism.^156^

### 6.2 Mitochondria and SOCE in heart failure with preserved ejection fraction (HFpEF)

CRU, formed by DHPR and RyR2, elicits the accumulation of CCMs adjacent to myofilaments, providing a coupling mechanism that enables Ca^2+^‐dependent myofilament activation, also referred to as excitation‐contraction coupling. NCX‐ and SERCA‐dependent dissipation of CCMs is a prerequisite for cardiac excitation‐contraction uncoupling. Therefore, the malfunction of the dissipation‐machinery of CCMs causes the retention of Ca^2+^ in the cytoplasm, resulting in HFpEF. Recent studies have demonstrated that SOCE and mitochondria contribute to HFpEF; SOCE improves the elevation of cytosolic Ca^2+^ and the development of cellular hypercontracture^157^; aldosterone/MR signalling enhanced SOCE is associated with increased diastolic [Ca^2+^]i that may inhibit the diastolic function of cardiomyocytes.^158^ The interplay between SOCE and mitochondria maintains Ca^2+^ entry during the diastolic period of cardiomyocytes, resulting in sustained elevation of cytosolic Ca^2+^ and HFrEF.

## 7 MITOCHONDRIA AND SOCE IN DIABETIC CARDIOMYOPATHY

**FIGURE 5:**
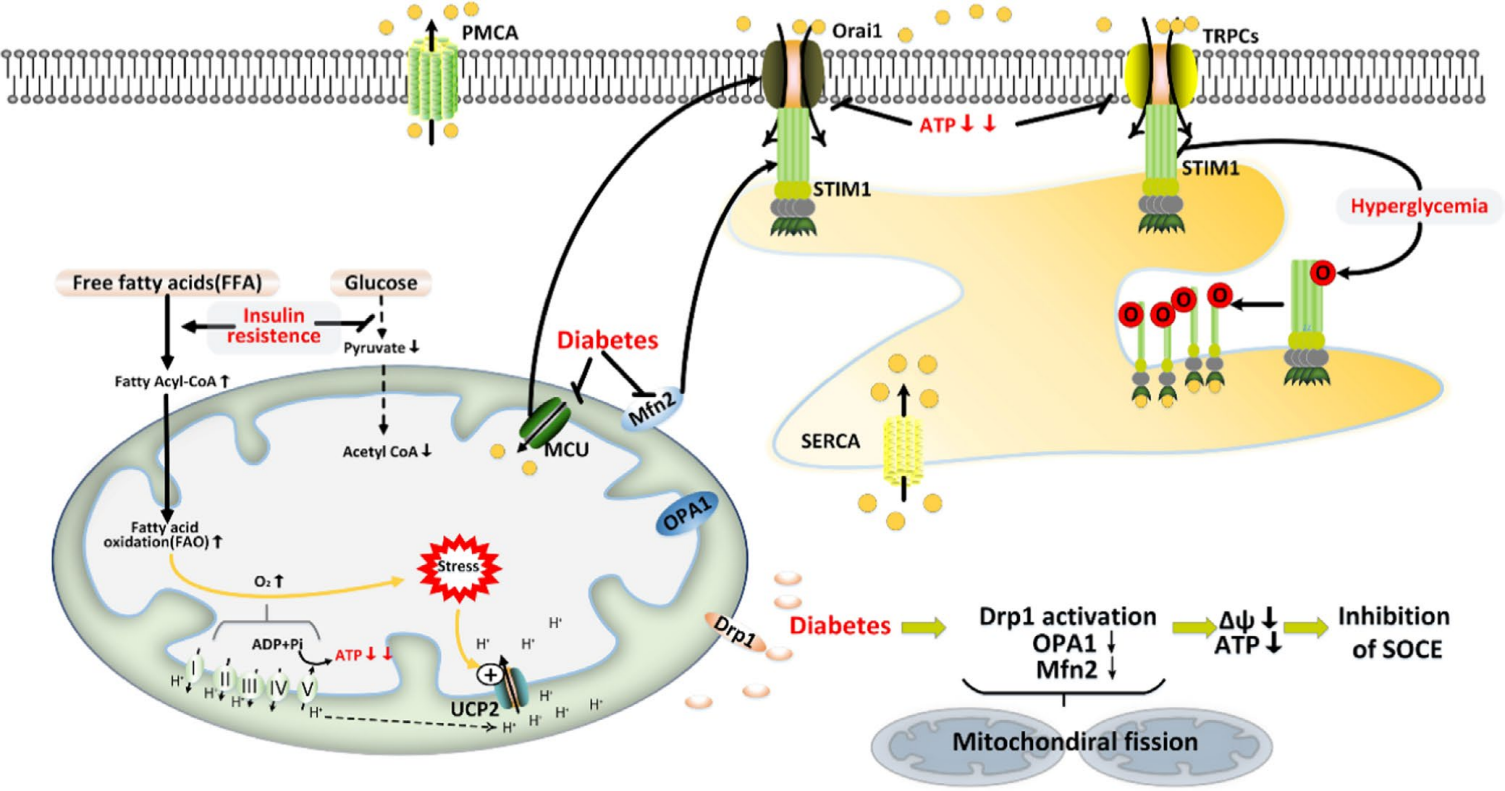
Mitochondria and SOCE are reciprocally regulated in diabetic cardiomyopathy. Hyperglycaemia mediated STIM1 O‐GlcNAcylation decrease SOCE activity. In diabetic cardiomyocytes, impairment of glucose metabolism and compensatory increased FFA metabolism increase UCP2 activity and subsequently decrease ATP production; due to altered protein levels and activity of mitochondrial dynamic‐related proteins (OPA1, Mfn2, and Drp1), the pro‐fission activity of mitochondria is highly active in diabetic cardiomyocytes, resulting in decreased Δψ and ATP content. Decreased ATP could not effectively buffer CCMs in the proximity of Ca^2+^ entry sits and subsequently facilitate Ca^2+^ dependent inactivation of SOCE. Moreover, reduced protein levels of Mfn2, a mitochondrial fusion protein, weaken the migration of STIM1 to SOCE channels, and ensuing SOCE activation in diabetic cells. Diabetic mouse hearts exhibit altered expression of MCU, which results in reduced mitochondrial Ca^2+^ uptake and subsequent attenuated SOCE activity. O denotes O‐GlcNAcylation

Diabetes is characterised by hyperglycaemia and insulin resistance. The attachment of O‐linked N‐acetylglucosamine to proteins (O‐GlcNAcylation) is observed under hyperglycaemic conditions. In cardiomyocytes, hyperglycaemia‐mediated STIM1 O‐GlcNAcylation decreases SOCE activity^10^, ^159^ (Figure 5). However, the potential role of insulin in SOCE activity still needs to be explored.^160^

[Ca^2+^]m uptake is vital for maintaining SOCE activity, and MCU is critical for inducing [Ca^2+^]m uptake.^102^, ^161^, ^162^ However, changes in the MCU members in the diabetic heart impair the capacity of [Ca^2+^]m uptake.^160^ In mice with diabetes, impairment of glucose consumption and a compensatory increase in free fatty acid levels promotes mitochondrial stress and activate UCP2, leading to the collapse of mitochondrial potential (Δψ) and reduced ATP production.^163^, ^164^, ^165^, ^166^ Since ATP can diffuse to the vicinity of SOCE channels to buffer the incoming Ca^2+^ counteracting the inhibitory effects of Ca^2+^ on SOCE activity, the paucity of ATP attenuates the SOCE activity in the diabetic heart.

With the development of diabetes, mitochondrial dynamic‐related proteins could be another determinant of SOCE activity. In diabetic cardiomyocytes, pro‐fusion proteins (OPA1 and Mfn2) are downregulated, and the pro‐fission protein (Drp1) is activated to promote mitochondrial fission, leading to decreased Δψ and reduced production of ATP.^167^, ^168^, ^169^, ^170^ Depolarized mitochondria and decreased ATP content can attenuate SOCE activity. Moreover, decreased Mfn2 expression is observed in diabetic cardiomyocytes.^168^, ^171^, ^172^ In addition to its pro‐fusion activity, Mfn2 is essential for STIM1 trafficking to ER‐PM junctions and subsequently promotes SOCE activity.^33^ Therefore, the reduced expression of Mfn2 in diabetes attenuates the activation of SOCE (Figure 5).

In brief, impaired mitochondrial Ca^2+^ handling, ATP insufficiency, and mitochondrial fragmentation hamper the opening of SOCE channels and reduce Ca^2+^ entry into diabetic cardiomyocytes (Figure 5).

## 8 MITOCHONDRIA AND SOCE ARE RECIPROCALLY REGULATED IN MYOCARDIAL IRI

Ischaemia‐reperfusion (I/R) is characterised by Ca^2+^ overload that causes IRI. Studies have shown that SOCE activity is enhanced following reperfusion and then mediated Ca^2+^ overload, suggesting that the SOCE event is a major contributor to IRI.^173^, ^174^ Inhibition of STIM1 alleviates H9C2 cell apoptosis by reducing calcium overload during hypoxic reperfusion.^175^ TRPC3 overexpressed mice exhibit increased apoptosis of cardiomyocytes in response to I/R, demonstrating the detrimental role of SOCE in IRI.^173^ SOCE inhibitors have been shown to alleviate cardiac injury following I/R. In both endothelial cells and cardiomyocytes, it has been found that gadolinium, SKF96365, and 2‐aminoethoxydiphenyl borate, all of which are inhibitors of SOCE, significantly attenuate Ca^2+^ overload during I/R process^176^, ^177^, ^178^; moreover, resveratrol pretreatment alleviates myocardial IRI by inhibiting STIM1‐mediated intracellular calcium accumulation.^174^

Transcription of Mfn2 is increased upon ROS insult during I/R, and increased protein levels of Mfn2 facilitate the aggregation of STIM1 at the site of the ER‐PM junction to open the SOCE channels.^179^ Arrhythmia is a common complication of IRI, and the contribution of SOCE to arrhythmias has received increased attention; disordered Ca^2+^ cycling and Ca^2+^ overload resulting from SOCE activation are pivotal for the initiation and recurrent cardiac arrhythmia during I/R. Orai‐mediated SOCE events contribute to the occurrence of atrial and ventricular arrhythmias following I/R.^27^, ^129^, ^141^, ^143^ In short, the SOCE event occurs during I/R and contributes to I/R‐induced Ca^2+^ overload and arrhythmias.

During I/R, SOCE is activated to induce Ca^2+^ overload in the cytosol.^173^, ^176^, ^177^ Then, the redundant Ca^2+^ transfers from the cytoplasm to the mitochondrial matrix in an MCU‐dependent manner.^105^, ^180^ Alternatively, upregulated protein levels of Mfn2 tether the mitochondria and ER together, facilitating a direct transfer of Ca^2+^ from the ER lumen to the mitochondrial matrix^119^, ^181^ (Figure 4). Thereafter, [Ca^2+^]m accumulation causes overproduction of mtROS and activation of apoptosis pathways.^182^, ^183^ In brief, I/R activates the SOCE‐[Ca^2+^]m‐mtROS pathway to deteriorate IRI. Intriguingly, I/R‐mediated mtROS generation can increase the sensitivity of IP3R to maintain empty ER Ca^2+^ stores, thus stabilising the opening of SOCE channels.^118^, ^183^

SOCE can mediate Ca^2+^ overload and promote mitochondrial fission in response to I/R. The increased [Ca^2+^]c during I/R activates calcineurin that targets drp1 (S637) for its de‐phosphorylation.^184^ In addition to calcineurin, CaMKIα, a downstream signal of [Ca^2+^]c, phosphorylates Drp1; both calcineurin and CaMKIα promote translocation of Drp1 to mitochondria to induce mitochondrial fission.^125^ These studies indicate that I/R activates the SOCE‐[Ca^2+^]c‐Drp1 signal to promote mitochondrial fission (Figure 4). ATP production is impaired during I/R, leading to diminished SERCA activity and ensuing relatively empty ER Ca^2+^‐stores^185^, ^186^; hence, impairment of ATP production may contribute to increased and sustained SOCE activity in response to I/R.

## 9 CONCLUSIONS

Mitochondria, the primary energy source for cardiomyocytes, occupy approximately 30% of the cardiomyocyte space. Apart from energy generation, mitochondria are major intracellular calcium stores, suggesting their potential role in Ca^2+^ regulation in cardiomyocytes. Numerous studies have revealed that the mitochondria and SOCE are reciprocally regulated. Nevertheless, the exact molecular mechanisms by which mitochondria determine SOCE and vice versa are still obscure. Herein, we discussed findings that intact respiring mitochondria are fundamental for the activation and maintenance of SOCE, and that Ca^2+^ entry induced by SOCE has recently been demonstrated to link mitochondrial metabolic functions with cellular signal transduction. Reciprocal regulation between mitochondria and SOCE is thus essential for cardiomyocyte homeostasis.

In different pathological states of the heart, SOCE is either enhanced or inhibited, and Ca^2+^ handling, dynamics, subcellular localization, and metabolism of mitochondria are dramatically altered. Thus, the communication between mitochondria and SOCE machinery is complex, subject to substantial alterations, and ultimately drives numerous cellular processes in cardiomyocytes. The interplay between mitochondria and SOCE may lead to either beneficial or detrimental effects in different pathological settings. The issues raised in this review may uncover the yet unrecognised interplay patterns between mitochondria and SOCE in different cardiac diseases. Our review provides potential targets for studying SOCE and mitochondria in the heart: blockade of SOCE may alleviate intracellular calcium overload and mitochondria‐related apoptosis; intervention of MCU‐dependent mitochondrial Ca^2+^ uptake, mitochondrial dynamics, and mitochondrial metabolism may counteract SOCE‐induced Ca^2+^ entry, protecting cardiomyocytes against cardiac challenges.

## CONFLICT OF INTEREST

The authors declare that they have no conflicts of interest.

## AUTHOR CONTRIBUTIONS

**Lingjun Zhu:** Conceptualization (supporting); Supervision (lead). **Jinliang Nan:** Conceptualization (lead); Writing‐original draft (equal). **Jiamin Li:** Writing‐original draft (equal). **Yinuo Lin:** Writing‐review & editing (equal). **Muhammad Saif Ur Rahman:** Writing‐review & editing (equal). **Zhengzheng Li:** Writing‐review & editing (equal).
